# Minority stress and sleep disturbance: How does anxiety mediate the relationship between stress and sleep for a sample of sexual minority men?

**DOI:** 10.1192/j.eurpsy.2022.2089

**Published:** 2022-09-01

**Authors:** J. Gibbs, R. Fusco

**Affiliations:** University of Georgia, School Of Social Work, Athens, United States of America

**Keywords:** Anxiety, Sexual Minority, Sleep Disturbance, Minority Stress

## Abstract

**Introduction:**

There is growing evidence that sexual minority men (e.g., gay, bisexual) experience lower sleep quality when compared to their heterosexual peers. Minority stress (e.g., discrimination, victimization) may account for these differences, however little is known about these relationships and how generalized anxiety may play a role in sleep disturbance.

**Objectives:**

Therefore, the aims of this study are to (a) understand the relationship between minority stress and sleep disturbance in a sample of sexual minority men, and (b) test whether these relationships are mediated by generalized anxiety.

**Methods:**

In 2020, 241 sexual minority men were recruited across a south-eastern state in the USA. Participants were asked to respond to scales assessing perceived social stress, minority stress constructs (i.e., internalized homophobia, experiences of harassment, microaggressions), generalized anxiety, and sleep disturbance. Linear regressions were used to test the relationship between minority stress and sleep disturbance controlling for perceived social stress and to test mediation by generalized anxiety.

**Results:**

Two minority stress constructs (experiences of harassment, and microaggressions) and perceived social stress were found to have a positive relationship with sleep disturbance. Generalized anxiety symptoms fully mediated the relationship between minority stress and sleep disturbance.

**Conclusions:**

Because sleep quality has a profound impact on health, findings from this study suggest the need for psychological intervention to improve sleep for sexual minority men. Given that generalized anxiety fully mediates the relationship between minority stress and sleep, targeted anxiety-based interventions have the potential to reduce sleep disturbance disparities between heterosexual and sexual minority men.

**Disclosure:**

No significant relationships.

